# Endoscopic ultrasound-guided short-cut jejunojejunostomy within a long biliary limb post pancreaticoduodenectomy to facilitate endoscopic retrograde cholangiography

**DOI:** 10.1055/a-2731-6332

**Published:** 2025-11-10

**Authors:** Sebastian Zundler, Daniel Klett, Timo Rath, Deike Strobel, Jürgen Siebler, Markus F. Neurath, Maximilian Waldner

**Affiliations:** 1 9171 Department of Medicine 1, University Hospital Erlangen, Erlangen, Germany

A 73 year-old patient with recurrence of pancreatic cancer presented with jaundice and cholangitis. He had received pylorus-preserving pancreaticoduodenectomy (PPPD) without adjuvant chemotherapy 7 months earlier. Two months earlier recurrence had been noted and palliative chemotherapy had been initiated.

During the previous 3 weeks, the patient had been admitted to two different external hospitals for cholangitis, where MRCP showed bilobar dilation of the intrahepatic bile ducts due to a central metastasis. In both external hospitals and at our center, ERCP had been attempted but failed due to a long and kinking biliary limb.

EUS-guided and percutaneous transhepatic biliary drainage were considered as alternative approaches but were deemed suboptimal due to ascites persisting despite drainage as well as separate anastomoses reported for the left and right hepatic ducts. Since a previous CT scan had shown a favorable hairpin loop-like configuration of the biliary limb and in regard of potential re-interventions, discussion of the situation with the patient led to the shared decision of preferentially attempting short-cut jejunojejunostomy within the biliary limb instead of ballon-assisted enteroscopy to facilitate subsequent ERCP ( Fig. 1 , Video 1 ).

**Fig. 1:**
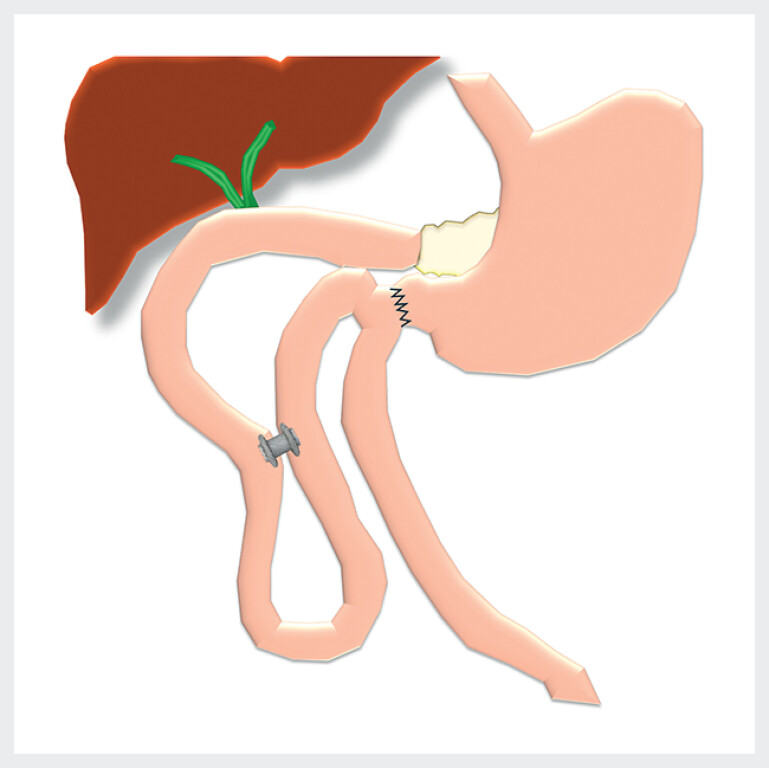
Scheme of post-surgical anatomy and jejunojejunostomy with a LAMS. LAMS, lumen apposing metal stent.

EUS-guided short-cut jejunojejunostomy within a long biliary limb post-PPPD to facilitate ERCP.Video 1

A colonoscope was introduced into the biliary limb as far as possible, and a guidewire was placed and used to position a 7 French nasobiliary catheter. The endoscope was exchanged for a linear echoendoscope, while 300 ml water were instilled via the catheter and peristalsis was blocked with butylscopolaminium and glucagon. A water-filled proximal portion of the biliary limb was identified on endoscopic ultrasound and a 15 mm × 10 mm lumen apposing metal stent (LAMS; Hot Axios, Boston Scientific) was placed in free-hand technique ( Fig. 2 **a** ).

**Fig. 2:**
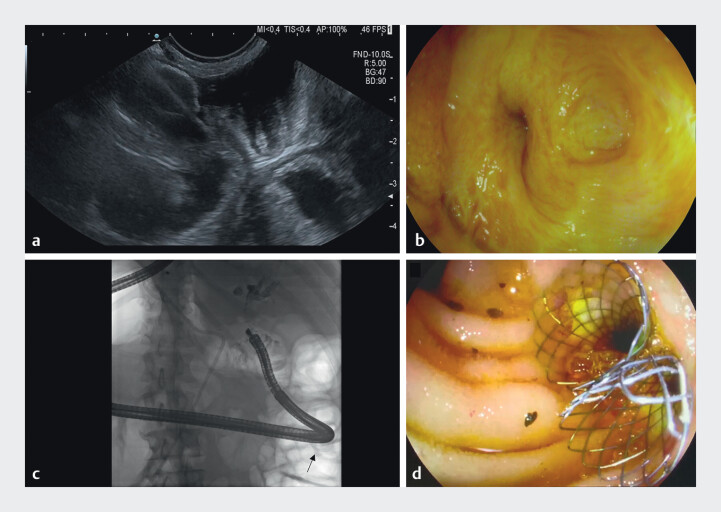
EUS-guided jejunojejunostomy and subsequent ERCP. **a** Following distension of the proximal part of the biliary limb with water, a LAMS was placed to create a short-cut jejunojejunostomy under EUS guidance. **b** Using this LAMS, biliodigestive anastomoses could be reached with a colonoscope. **c** Fluoroscopic image showing a cholangiogram as well as the passage of the colonoscope through the LAMS (arrow). **d** Uncovered metal stents were placed in both hepatic ducts. LAMS, lumen apposing metal stent.

In a second intervention, the LAMS was dilated to 15 mm and a colonoscope was advanced through the LAMS (at 70 cm) up to the biliodigestive anastomoses (at 110 cm; Fig. 2 **b** and **c** ). Cholangiography confirmed strictures and 40 mm × 10 mm uncovered metal stents were placed in both hepatic ducts ( Fig. 2 **d** ).

Adequate drainage was achieved and no complications or adverse events occurred.

Endoscopy_UCTN_Code_TTT_1AS_2AK

## Notes

Endoscopy E-Videos https://eref.thieme.de/e-videos
*E-Videos* is an open access online section of the journal *Endoscopy* , reporting on interesting cases and new techniques in gastroenterological endoscopy. All papers include a high-quality video and are published with a Creative Commons CC-BY license. Endoscopy E-Videos qualify for HINARI discounts and waivers and eligibility is automatically checked during the submission process. We grant 100% waivers to articles whose corresponding authors are based in Group A countries and 50% waivers to those who are based in Group B countries as classified by Research4Life (see: https://www.research4life.org/access/eligibility/ ). This section has its own submission website at https://mc.manuscriptcentral.com/e-videos .

